# Transcriptome analysis in *Hevea brasiliensis* latex revealed changes in hormone signalling pathways during ethephon stimulation and consequent Tapping Panel Dryness

**DOI:** 10.1038/s41598-018-26854-y

**Published:** 2018-05-31

**Authors:** Pascal Montoro, Shuangyang Wu, Bénédicte Favreau, Eva Herlinawati, Cécile Labrune, Marie-Laure Martin-Magniette, Stéphanie Pointet, Maryannick Rio, Julie Leclercq, Sigit Ismawanto, Kuswanhadi

**Affiliations:** 1 CIRAD, UMR AGAP, F-34398 Montpellier, France; 2 AGAP, Univ Montpellier, CIRAD, INRA, Montpellier SupAgro, Montpellier, France; 3 CAS Key Laboratory of Genome Sciences and Information, Beijing Institute of Genomics, Chinese Academy of Sciences, Beijing, 100101 China; 4 University of Chinese Academy of Sciences, Beijing, 100049 China; 5 Indonesian Rubber Research Institute, Sembawa Research Centre, Palembang, Indonesia; 6 Institute of Plant Sciences Paris Saclay IPS2, CNRS, INRA, Université Paris-Sud, Université Evry, Université Paris-Saclay, Bâtiment 630, 91405 Orsay, France; 7 Institute of Plant Sciences Paris-Saclay IPS2, Paris Diderot, Sorbonne Paris-Cité, Bâtiment 630, 91405 Orsay, France; 8 UMR MIA-Paris, AgroParisTech, INRA, Université Paris-Saclay, 75005 Paris, France

## Abstract

Tapping Panel Dryness (TPD) affects latex production in *Hevea brasiliensis*. This physiological syndrome involves the agglutination of rubber particles, which leads to partial or complete cessation of latex flow. Latex harvesting consists in tapping soft bark. Ethephon can be applied to stimulate latex flow and its regeneration in laticifers. Several studies have reported transcriptome changes in bark tissues. This study is the first report on deep RNA sequencing of latex to compare the effect of ethephon stimulation and TPD severity. Trees were carefully selected for paired-end sequencing using an Illumina HiSeq 2000. In all, 43 to 60 million reads were sequenced for each treatment in three biological replicates (slight TPD trees without ethephon stimulation, and slight and severe TPD trees with ethephon treatment). Differentially expressed genes were identified and annotated, giving 8,111 and 728 in response to ethephon in slight TPD trees and in ethephon-induced severe TPD trees, respectively. A biological network of responses to ethephon and TPD highlighted the major influence of metabolic processes and the response to stimulus, especially wounding and jasmonate depression in TPD-affected trees induced by ethephon stimulation.

## Introduction

Natural rubber produced by *Hevea brasiliensis* accounts for more than 40% of rubber consumption worldwide. With more than 10 million hectares, rubber cultivation and its industry play an important socio-economic role in rubber producing countries. Natural rubber is synthesized in laticifers, which form an articulated network of anastomosed latex cells. Latex containing natural rubber is collected by tapping the bark of rubber trees. Latex is the cytoplasm of laticifers. Ethylene induces several metabolic pathways in latex. For certain *Hevea* clones, the application of ethephon, an ethylene releaser, stimulates latex regeneration between two tappings and latex flow after tapping, hence latex production. The growing demand for natural rubber prompts farmers to use quick-starter clones and intensive harvesting systems, which have led to increasingly large losses in rubber production. The physiological syndrome called Tapping Panel Dryness (TPD) is responsible for an estimated 12 to 20% loss in rubber production^1^, which could increase in a context of climate change.

Environmental and harvesting stresses can lead to oxidative stress in laticifers and consequently to TPD induction^2^. A lutoid is a single-membrane microvacuole with lysosomal characteristics. Lutoidic NAD(P)H oxidase is a major source of superoxide anion species in latex (Chrestin *et al*. 1984). This production of reactive oxygen species (ROS) induces peroxidatic degradation of the unsaturated lipids of the membrane. Such lutoid instability leads to the release of lutoid proteins in cytosol and promotes rubber particle agglutination^3^. Under exacerbated conditions, an irreversible type of TPD, called brown bast (BB), occurs. This form is related to deformation of the bark due to thylosoid formation, lignified gum, and abnormal division of parenchyma cells, and may be related to a cyanogenesis process^4,5^. These two types of TPD were recently called ROS-TPD and BB-TPD^6^. TPD, trunk phloem necrosis, brown bast, dry bark, etc. show the same anatomical symptom^4^. However, these syndromes might be related to different stress origins such as rootstock-scion incompatibility^7^, soil compaction, and environmental or harvesting stress^8^.

Numerous molecular studies have led to the identification of TPD-regulated genes, such as *HbMyb1, HbTOM20* and *HbTCTP1*^3,9–16^. Differential transcript abundance was observed for twenty-seven candidate genes, related to TPD occurrence, in latex and phloem tissues for ROS-scavenging, ethylene biosynthesis and signalling genes^6^. The technologies used in these previous studies led to partial identification of genes associated with TPD occurrence. More recently, the technology of RNA sequencing has opened up new perspectives for a full comprehensive analysis of TPD through a transcriptome comparison between TPD-free and TPD-affected trees. Given the stoppage in latex flow, deep sequencing of RNA has focused solely on bark tissues of severe TPD-affected trees. These analyses notably revealed a down-regulation of genes related to rubber biosynthesis^17^ as well as jasmonate production^18^. A few studies have attempted to analyse latex from early TPD-affected trees. The first one revealed post-transcriptional regulation with both RNA degradation and a shift in miRNA biogenesis^19^. The second suggested differential expression of proteins related to rubber biosynthesis, the reactive oxygen metabolism and cell apoptosis^20^.

This paper sets out to provide new insight into the molecular regulatory mechanism of ethephon-induced TPD in latex and to identify a set of differentially expressed genes associated with ethephon-induced TPD occurrence. The study identified a set of differentially expressed genes associated with TPD occurrence. Several earlier publications tried to study TPD, but at the last stage of TPD. They therefore only obtained 100% TPD-affected trees and had to study bark tissue. The novelty of our study is to carefully monitor the onset of the syndrome and manage to select trees with slight and severe TPD symptoms still able to produce some latex. Regular monitoring of the dry cut length (DCL) on the tapping panel revealed that all the trees had a variation in DCL value^6^. High latex viscosity in a localized part of the bark reduces latex flow. This transient symptom concerns a small proportion of the tapping cut. In contrast, the ROS-TPD type resulting from the physiological fatigue of laticifers concerns a larger share of the tapping cut, which increases regularly over the tapping period and requires a tapping rest of several months for total recovery. Ethephon treatment accelerates the latex metabolism and induces earlier and greater TPD occurrence, especially for rubber clone PB 260 with a high latex metabolism^6^. Firstly, the effect of ethephon was analysed on slight TPD-affected trees. Secondly, slight and severe TPD-affected trees subjected to ethephon stimulation were compared. RNA sequencing was carried out in three biological replicates and a differential expression analysis was carried out. This study identified 728 differentially expressed genes associated with ethephon-induced TPD, 272 of which were specifically regulated by TPD and not by ethephon treatment. When ethephon-induced TPD became established, an overall decrease in gene expression was observed, except for genes related to the GO “stimulus response” term, particularly those related to the wounding and jasmonic acid pathways.

## Results

### RNA sequencing and mapping on the reference transcriptome

**Figure 1:**
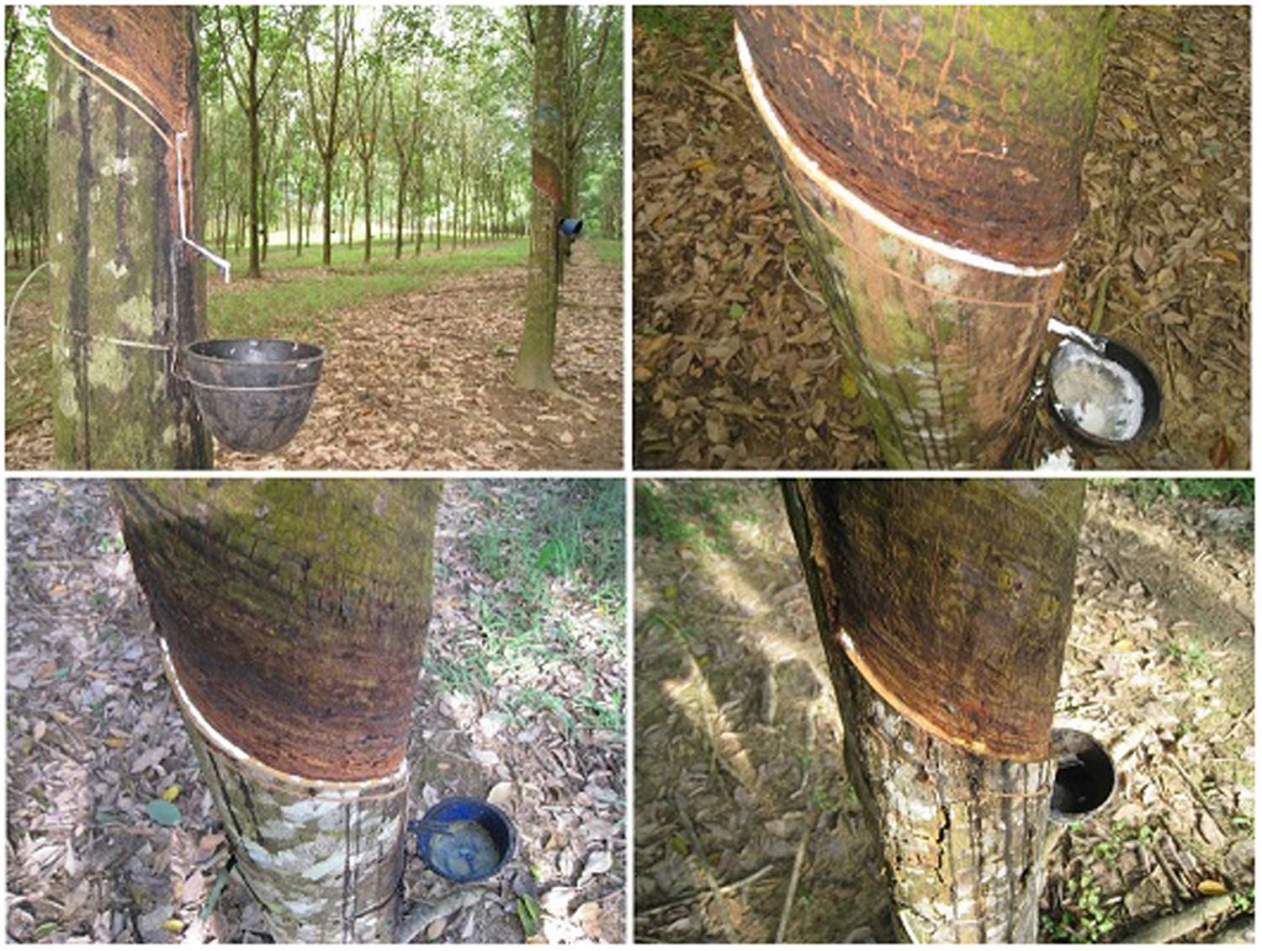
Gradient of Tapping Panel Dryness (TPD) symptoms in *Hevea brasiliensis*. (**A**) Normal latex flow along the entire tapping cut. (**B**) Tapping cut of a healthy tree. (**C**) Tapping cut of a slight TPD-affected tree. (**D**) Tapping cut of severe TPD-affected tree.

Latex flows out from laticifer vessels after tapping soft bark. Latex drops appear instantly on the tapping cut. The dry cut length (DCL) was recorded just after tapping and before latex flowed along the cut. Most trees showed a partial DCL ranging from 0 to 100% (Fig. 1). Slight TPD-affected trees (DCL < 25%, Fig. 1B) were found in trees that were treated and not treated with ethephon. By contrast, severe TPD-affected trees (DCL > 75%, Fig. 1D) were identified only in ethephon-treated trees. The DCL was not strictly stable for each tree due to localized and partial TPD occurrence on the tapping panel (Suppl. Data 1). For that reason, trees were classified as slight or severe TPD depending on the mean DCL over the last 12 months of tapping before sampling (Table 1).

**Table 1:** Phenotype of trees selected for the RNA sequencing experiment.

Class of trees	Ethephon treatment (%)	DCL (%)	Tree phenotype
Control, no-ET	0	4	Slight TPD
	0	4	Slight TPD
	0	4	Slight TPD
Slight TPD, ET-treated	2.5	6	Slight TPD
	2.5	15	Slight TPD
	2.5	16	Slight TPD
Severe TPD, ET-treated	2.5	95	Severe TPD
	2.5	80	Severe TPD
	2.5	81	Severe TPD

Trees were tapped every two days for 3 years. Ethephon stimulation was applied 12 times a year. The dry cut length (DCL) was monitored monthly. Trees were considered as slightly affected when DCL < 25% and severely affected by TPD when DCL > 75%.

RNAs from the latex of non ethephon-treated trees (control), and both slight and severe ethephon-induced TPD trees were paired-end sequenced using an Illumina HiSeq 2000. In all, 43 to 60 million reads were obtained for each oriented TRUSeq library (Suppl. Data 2). Trimmed reads were mapped on the rubber clone PB 260 reference transcriptome^21^. Of the 86,941 contigs, 72,910 were previously annotated by BLASTX. Reads were counted for the 86,941 contigs.

### Identification of differentially expressed genes in response to ethephon and TPD severity

A differential analysis was performed for the 86,941 contigs (Suppl. Data 3). Given the importance of small changes in RNA expression^22^, differentially expressed genes (DEGs) were selected when their FDR adjusted *p-value* was lower than 0.05. In latex, 8,111 DEGs were detected in response to ethephon stimulation with a similar number of over-expressed (3883) and under-expressed (4,228) genes (Suppl. Data 4, Table 2).

**Table 2:** Effect of ethephon and severity of TPD occurrence on the number of differentially expressed genes (DEGs).

Level of expression	Number of DEGs		
	Ethephon vs control (slight TPD trees)	Severe vs slight TPD (ethephon-treated trees)	TPD-specific (ethephon-treated trees)
Over-expressed	3883	97	28
Under-expressed	4228	631	244
Total	8111	728	272

Ethephon-induced TPD-affected trees were identified in the set of ethephon-treated trees only. An analysis of slight and severe TPD-affected trees from the same set of ethephon-treated trees revealed 728 DEGs consisting of 97 over- and 631 under-expressed genes (Suppl. Data 5). Unlike DEGs identified in response to ethephon, 6 times more under-expressed genes than over-expressed genes were identified in trees with severe TPD. The expression of 456 of these ethephon DEGs (over- and under-expressed) was accentuated in response to TPDfrom ethephon to TPD for both over-expressed and under-expressed genes by ethephon were more highly expressed under TPD as well as for under-expressed genes. Of the 728 DEGs associated with severe TPD, expression of 272 genes was not significantly changed by ethephon treatment in slight TPD trees (Suppl. Data 6). The 272 DEGs were mostly under-expressed (244) rather than over-expressed (28).

**Figure 2:**
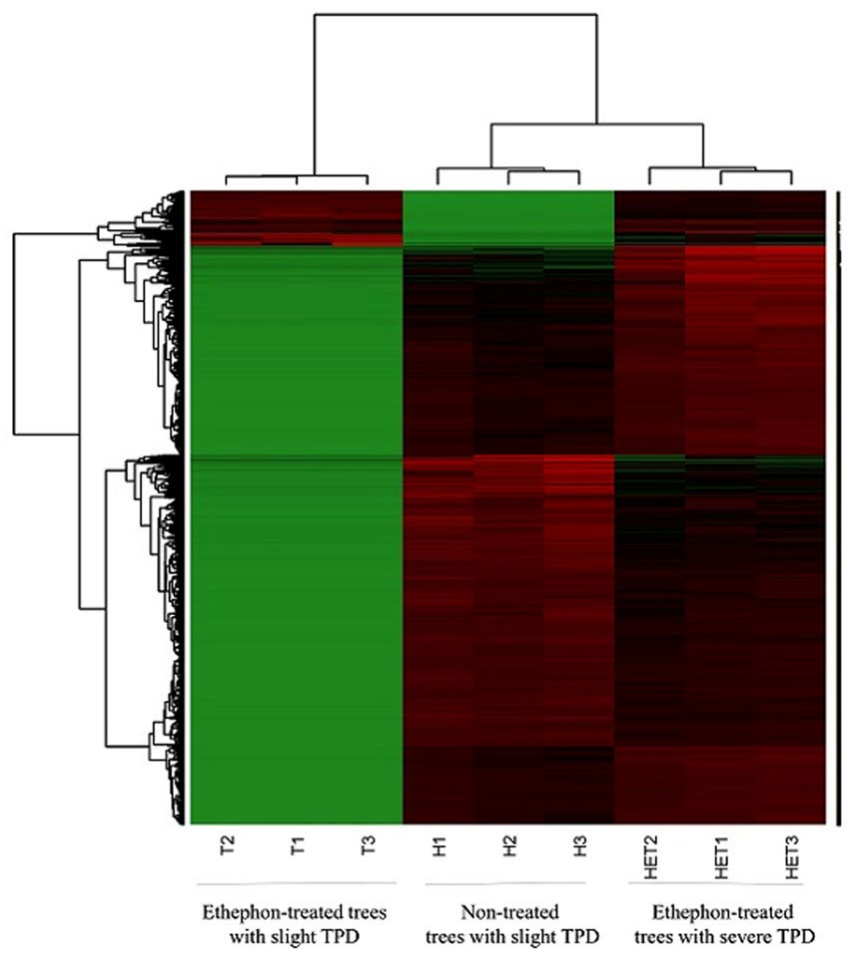
Heatmap hierarchical clustering of the 8,300 contigs based on the count distribution of the nine samples (three biological replicates × three treatments). The treatments are latex from control, slight TPD trees with ethephon stimulation, and latex from severe TPD-affected trees with ethephon stimulation.

A principal component analysis (PCA) of the read data was carried out for 8,111 DEGs. The PCA highlighted the variation between the three types of samples (control, slight TPD trees treated with ethephon, severe TPD-affected trees treated with ethephon) (Suppl. Data 7). The first two components explained 92% of the variability between samples, discriminating between slight and severe TPD trees on the first axis (62%), and slight TPD-affected trees treated or not with ethephon (control), on the second axis (30%). Heatmap hierarchical clustering of the DEGs was used to visualize count distribution in the nine samples (Fig. 2). The samples were clustered by treatment, with two main clusters representing severe and slight TPD trees, the latter being sub-clustered according to ethephon treatment. The heatmap highlighted specific gene expression patterns for each of the three treatments. Latex samples from control trees without ethephon treatment had a large number of over-expressed genes, while ethephon-treated latex samples revealed overall up-regulation of genes, especially for genes under-expressed in the treatment without ethephon. Conversely, gene expression in latex from ethephon-induced severe TPD trees was mainly under-expressed.

### Gene ontology, classification and biological network of response to ethephon and TPD severity

In order to improve the annotation of DEGs, contig sequences were blasted using scaffolds from published genomes as the target^23^. An InterProScan was performed with corresponding peptide sequences from the rubber reference genome. InterProScan GOs were merged to annotation in order to run Blast2GO analyses (Suppl. Data 8). The first two levels of gene ontology (GO) classification were presented for up- and under-expressed genes in response to ethephon and TPD severity (Table 3). A large number of ethephon-regulated DEGs was identified (>500) for the cellular process, metabolic process, binding and catalytic activity GO terms. More GO terms were counted for ethephon over-expressed genes compared to under-expressed genes, except for the “response to stimulus”, “single organism process”, “membrane” and “membrane part” terms. For all the ethephon-regulated genes, most of the GO terms had more than 100 DEGs except for “response to stimulus”, “membrane part”, “organelle part”, “nucleic acid binding transcription factor activity” and “transporter activity”. Ethephon under-expressed genes had fewer GO terms. For ethephon-induced TPD-related DEGs, GO annotation showed that most of the ethephon over-expressed genes participated in the biological process and molecular function. Level 2 GO terms belonged to “biological regulation”, “cellular process”, “metabolic process”, “regulation of biological process”, “binding and catalytic activity”. TPD under-expressed genes were related to most of the GO terms except “nucleic acid binding transcription factor activity” and “transporter activity” in the molecular function group.

**Table 3:** Number of Differentially Expressed Genes (DEGs) for the first two levels of Gene Ontology (GO) for over- and under-expressed genes, in response to ethephon and severity of ethephon-induced TPD.

GO Name		Number of DEGs			
		Ethephon vs control		Severe vs slight TPD	
Level 1	Level 2	Over-expressed	Under-expressed	Over-expressed	Under-expressed
Biological process	biological regulation	187	135	11	37
	cellular process	740	472	24	110
	localization	111	99	4	35
	metabolic process	768	571	28	143
	regulation of biological process	182	129	11	37
	response to stimulus	0	86	4	27
	single-organism process	305	317	9	84
Cellular component	cell part	355	158	2	48
	cell	355	158	2	48
	membrane part	72	95	0	22
	membrane	121	149	4	39
	macromolecular complex	209	48	0	14
	organelle	257	99	2	34
	organelle part	96	35	0	10
Molecular function	binding	912	768	27	183
	catalytic activity	589	583	23	143
	nucleic acid binding transcription factor activity	0	0	6	0
	transporter activity	0	0	3	0

**Figure 3:**
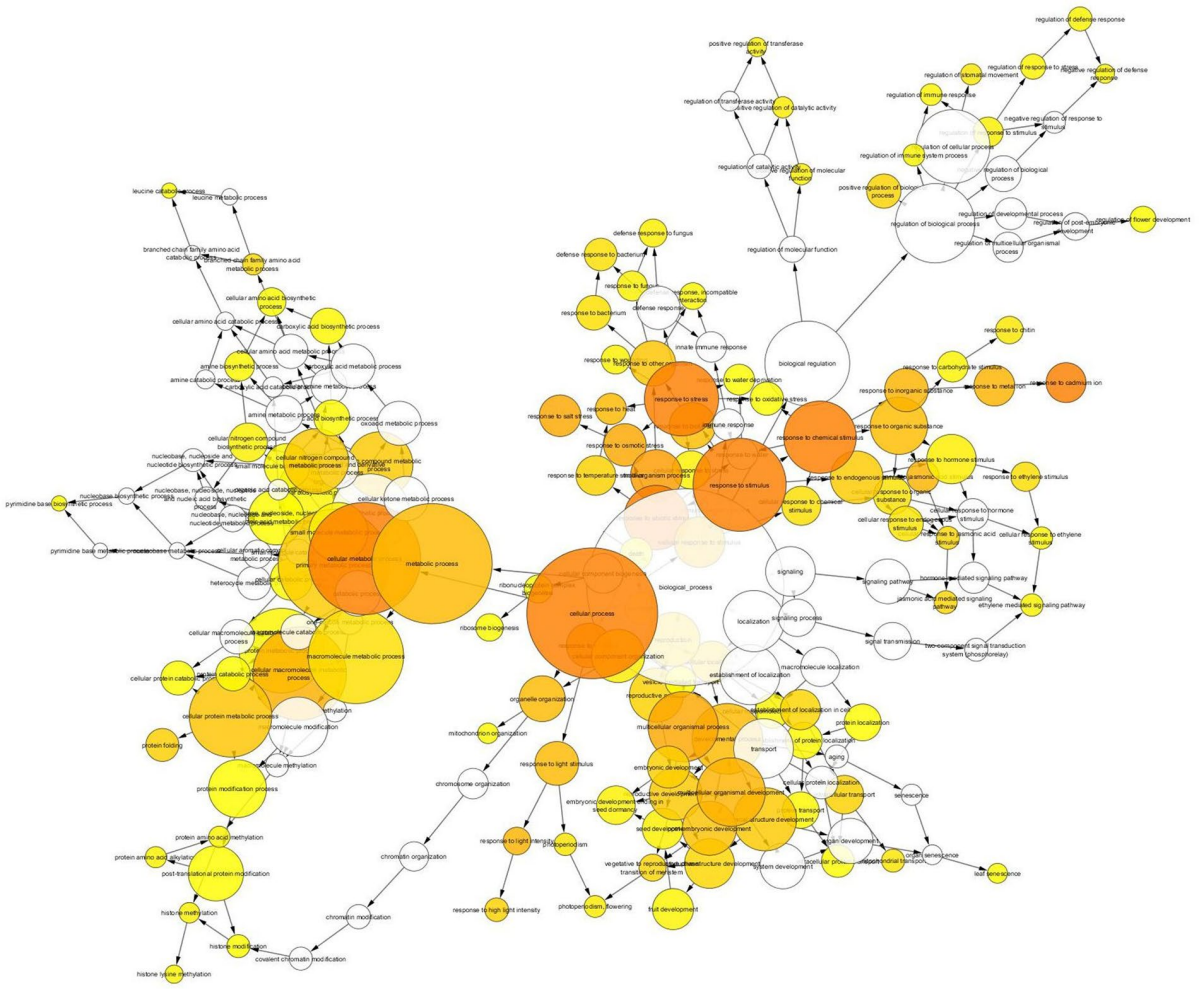
BINGO network analysis using Cytoscape 3.4.0 for DEGs over-expressed by ethephon. The colour of the nodes represents the significance of the over-representation, with a colour scale ranging from yellow at a p-value of 0.05 to dark orange at a p-value corresponding to 5 orders of magnitude smaller than the significance level (10^−5^ × 0.05).

**Figure 4:**
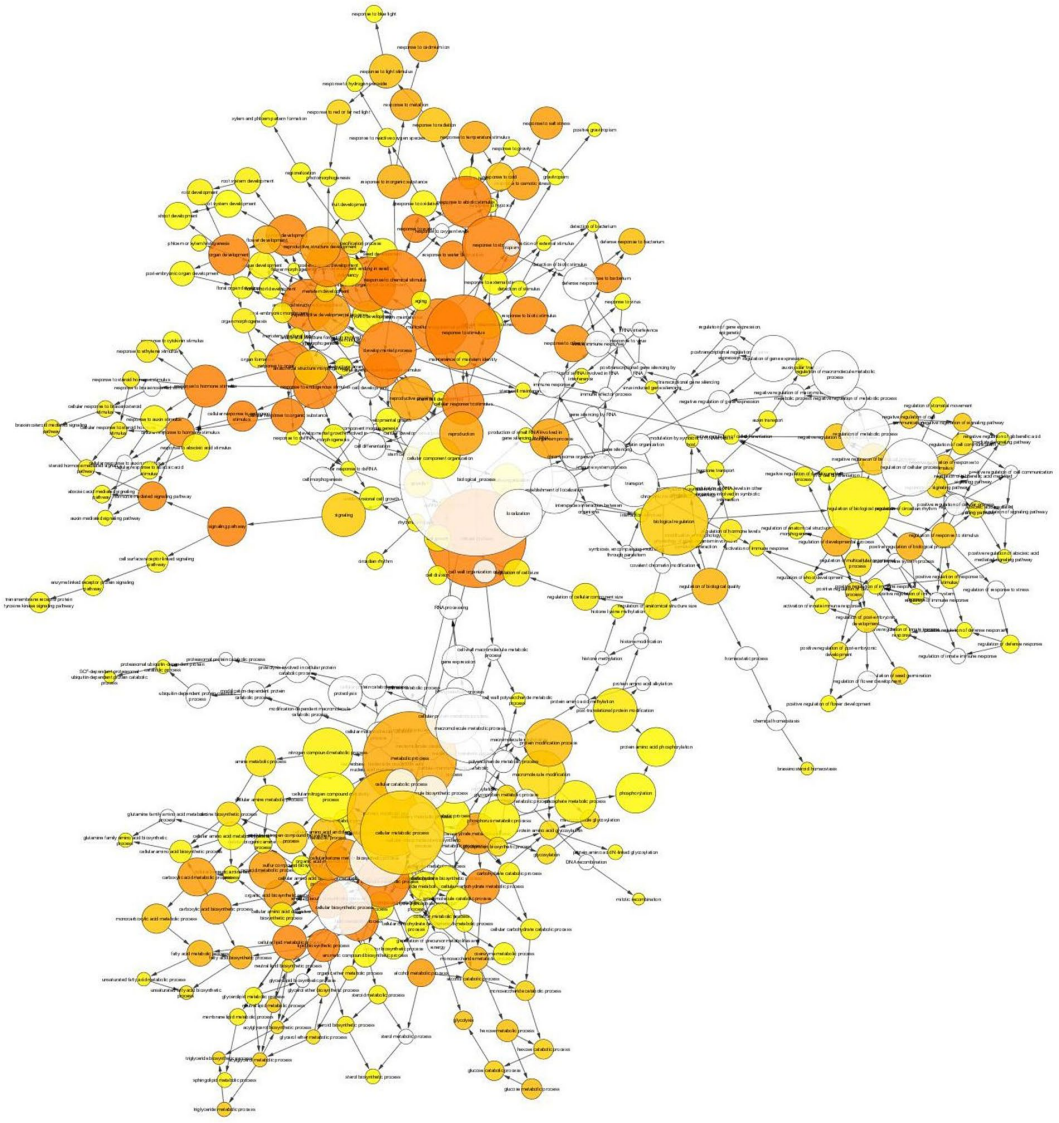
BINGO network analysis using Cytoscape 3.4.0 for DEGs under-expressed by ethephon. The colour of the nodes represents the significance of the over-representation, with a colour scale ranging from yellow at a p-value of 0.05 to dark orange at a p-value corresponding to 5 orders of magnitude smaller than the significance level (10^−5^ × 0.05).

**Figure 5:**
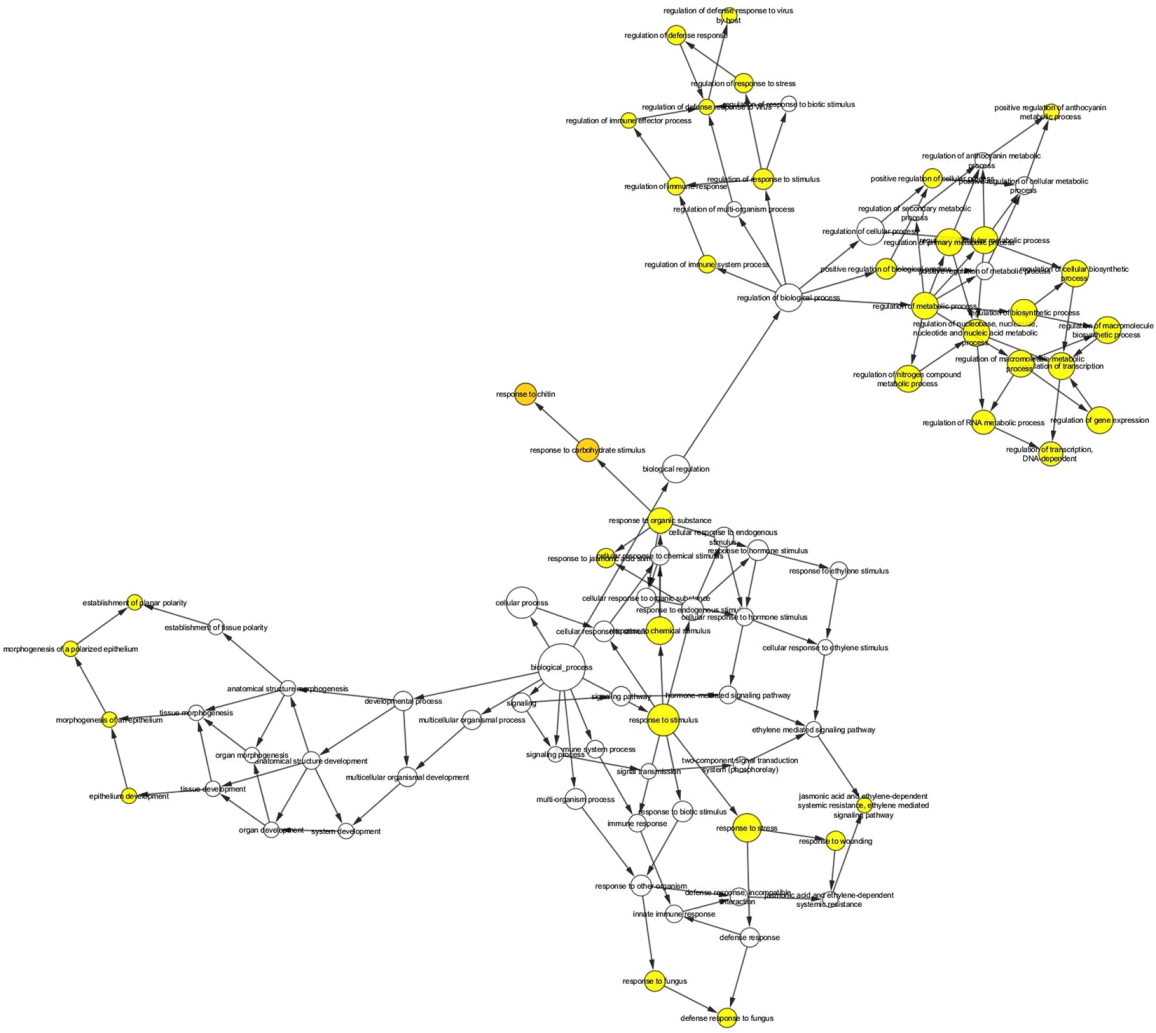
BINGO network analysis using Cytoscape 3.4.0 for DEGs over-expressed in severe TPD. The colour of the nodes represents the significance of the over-representation, with a colour scale ranging from yellow at a p-value of 0.05 to dark orange at a p-value corresponding to 5 orders of magnitude smaller than the significance level (10^−5^ × 0.05).

**Figure 6:**
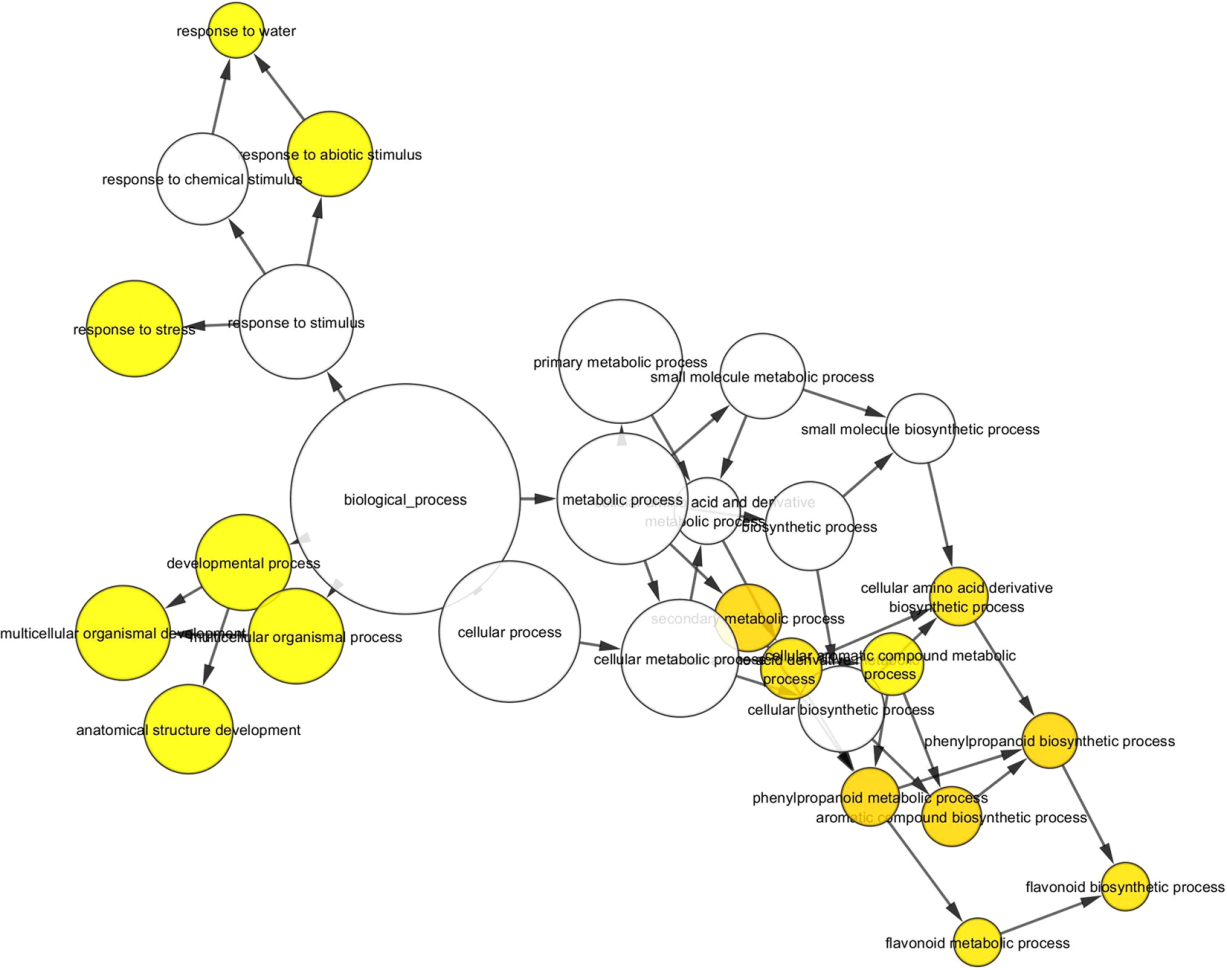
BINGO network analysis using Cytoscape 3.4.0 for DEGs under-expressed in severe TPD. The colour of the nodes represents the significance of the over-representation, with a colour scale ranging from yellow at a p-value of 0.05 to dark orange at a p-value corresponding to 5 orders of magnitude smaller than the significance level (10^−5^ × 0.05).

A network analysis was carried out for the four DEG groups. For DEGs over-expressed by ethephon, biological processes including “metabolic process” (primary metabolism, macromolecule, protein, etc.), and “response to stimulus” (hormone, abiotic, biotic, oxidative stress) were overrepresented (Fig. 3, Suppl. Data 9). For DEGs under-expressed by ethephon, similar functions were reversely regulated (Fig. 4, Suppl. Data 10). For DEGs over-expressed in severe ethephon-induced TPD, a much smaller number of genes was significantly annotated (Fig. 5, Suppl. Data 11). A few DEGs, related to response stimulus with, in particular, jasmonic acid, chitin, wounding and fungus, were still activated. Some genes related to the regulation of the biological process were also activated, in particular the regulation of response to stimulus and metabolic processes. For the last ethephon-induced TPD under-expressed DEG group, a very small number of DEGs was classified: GO terms were related to “response to stimulus” (stress and abiotic), “developmental process” and “secondary metabolic process” (phenylpropanoid metabolic process) (Fig. 6, Suppl. Data 12).

### Identification and regulation of DEGs encoding transcription factors in response to ethephon and severity of TPD

Transcription factors are crucial regulators of gene expression. They were extracted from the two classes of ethephon-induced TPD treatments in Supplementary Data 13 and 14, respectively. They were classified according to their membership of the 13 most represented families of transcription factors (Table 4). The top five families in response to ethephon treatment were Ethylene Response Factors (ERF; 39 contigs), followed by Auxin Response Factor (ARF; 33 contigs), WRKY (31 contigs), Homeobox superfamily (31 contigs) and bHLH (29 contigs). In addition, WRKY (4 contigs), Myb (2 contigs), bHLH (2 contigs), ERF (1 contig) and MYC (1 contig) were the top ten most over-expressed genes in response to ethephon treatment (Supplementary Data 13). For each family of transcription factors, some genes were over- or under-expressed at a similar level, except for ARF, homeobox and MADS box, which had more under-expressed genes (24/33, 34/31 and 8/12, respectively).

**Table 4:** Main transcription factor families regulated in response to ethephon and severity of ethephon-induced TPD.

Transcription factor family	Ethephon vs control			Severe vs slight TPD		
	Total	Up	Down	Total	Up	Down
ERF	39	23	16	3	1	2
ARF	33	9	24	3	0	3
WRKY	31	16	15	4	4	0
Homeobox	31	7	24	5	0	5
bHLH	29	13	16	2	0	2
MYB	21	10	11	6	1	5
MADS box	12	4	8	1	0	1
TCP	12	5	7	2	0	2
GATA	11	5	6	1	0	1
MYC	9	4	5	1	0	1
HSF	7	4	3	2	0	2
NAC	4	1	3	1	0	1
GRAS	3	0	3	2	2	0
Other	74	32	42	17	3	14
Total	316	133	183	50	11	39

The comparison of slight and severe ethephon-induced TPD trees revealed a dramatic drop in the number of regulated transcription factors from 316 to 50 (Table 4). The under/over-expressed transcription factor ratio increased from 1.38 (183/133) in slight TPD-affected to 3.54 (39/11) in severe TPD-affected trees, highlighting a general decrease in plant signalling. Most of the TF families were under-expressed, except the GRAS family, which belongs to the SCARECROW (SCR) subfamily. They were identified in latex but were reversely regulated for slight TPD-affected (3 under-expressed) and severe TPD trees (2 over-expressed).

## Discussion

This study reports on the first deep RNA sequencing in the latex of ethephon-induced TPD-affected trees. It was made possible by the selection of slight and severe TPD-affected trees producing latex, based on the monitoring of TPD occurrence in a large panel of trees for 3 years. Latex from TPD trees was collected just before the tapping cut became totally dry. Several other studies were previously carried out on latex from healthy trees only, notably to understand molecular mechanisms related to rubber production^21,24–28^. With regard to TPD, some gene expression markers, such as *HbMyb1, HbTOM20* and *HbTCTP1*, have been identified through differential display RT-PCR, microarrays and suppression subtractive hybridization^9–11,15,29^. The expression of six *MYB* genes was changed in ethephon-induced TPD-affected trees (one over-expressed and five under-expressed). In contrast, none of the other candidate genes was associated with TPD, neither *HbTOM20* (2 contigs (CL1183Contig1 and CL1183Contig4) not expressed in latex) nor *HbTCTP1*, despite the high expression of one of the two contigs, CL1Contig21026 (107-142 reads) and CL1Contig7138 (41881-52466 reads). This variability in gene expression markers reveals that some candidate genes are associated with either clonal response or certain environmental or experimental conditions, and are not directly related to the biological process studied. In addition, these technologies provided a partial and a-priori view of transcriptome expression. More recently, high-throughput sequencing was used to describe global gene expression changes in bark^17,18^. Ethephon-induced TPD resulted in an overall decrease in gene expression, which was less in latex (631 DEGs) than in bark (8,137 and 22,577 DEGs)^17,18^. The down-regulation of some genes related to rubber biosynthesis in bark^17,18^ was confirmed at protein level^20^. Bark tissue consists of several cellular types (phloem, laticifer, vascular rays, sclereid). The larger number of over-expressed DEGs in bark, compared to latex, suggests that either at least one bark tissue type other than laticifers might maintain a certain level of metabolic activity and gene expression, or the greater stringency in this study led to fewer DEGs being selected.

Ethephon is applied to the tapping cut of certain rubber clones to stimulate latex metabolism and prolong latex flow, and consequently latex production^30^. Rubber clone PB 260, studied in this paper, has an intrinsic high latex metabolism^6^. For this clone, ethephon application can accelerate TPD occurrence: more than 60% of ethephon-treated trees were severely affected by TPD after 15 months of stimulation, while a small number of trees had a partial dry cut without ethephon treatment^6^. Several reports have revealed the dramatic change in gene expression in latex from ethephon-treated trees using RNA sequencing (8111 DEGs for clone PB 260 (this study), 10216 DEGs for clone PR 107^24^, 509 DEGs for clone Reyan7-33-97^23^), and cDNA microarrays (163 DEGs^31^). A proteomics analysis of ethylene-stimulated rubber latex identified 143 and 404 ethylene responsive latex proteins using mass spectrometry from 2-DE and DIGE gels, and iTRAQ, respectively^32^. The present study actually revealed only 3,883 over-expressed DEGs, mostly involved in “cellular process”, “metabolic process”, “binding” and “catalytic activity”. Wang and co-workers concluded that ethylene improves carbohydrate catabolism and energy production but not rubber biosynthesis itself directly^32^, confirming rapid acceleration of the glycolytic pathway supplying precursors for the biosynthesis of IPP, as suggested by Liu and co-workers too^24^.

Ethylene biosynthesis and signalling pathways are particularly affected by ethephon stimulation. Following the first isolation of ethylene biosynthesis genes^33,34^, a genome-wide analysis showed that the *Hevea* genome activated 8 genes encoding S-adenosyl-L-methionine synthase (SAMS), 14 genes encoding 1-aminocyclopropane-1-carboxylic acid (ACC) synthase (ACS) and 16 genes encoding ACC oxidase (ACO)^23^. Although 7 ACS and 8 ACO contigs were identified in the present RNA sequencing analysis, a small number of counts confirmed previous assumptions of low ethylene production in latex^23,33^. By contrast, latex cells are well-equipped for ethylene perception and signalling. A large number of ethylene receptors were expressed: 7 contigs in clone PB 260 (present work, Suppl. Data 4) and 4 genes in clone Reyan7-33-97^23^. The reference genome has 181 Ethylene Response Factors (ERF)^23^, while only 91 have been classified and functionally verified^21^. For rubber clone PB 260, 27 and 39 DEGs encoding ERF were identified in latex by Piyatrakul and co-workers^21^ and in the present study, respectively. In clone Reyan7-33-97, 21 DEGs were reported^23^. These results suggest a regulation of ethylene responsive genes in latex by ERFs in these rubber clones. Several members of three ERF groups (I, VII, VIII) are highly expressed in latex^21^. HbERF-II and HbERF-VIIIa deduced proteins contained the repressor EAR motif. Their high expression under normal conditions suggests a certain negative control of ethylene response. By contrast, their lowering in TPD-affected trees is expected to activate ethylene responsive genes. This deregulation could be the source of latex cell dysfunction. Several members of HbERF-IX are assumed to be essential integrators of complex hormonal signalling pathways in *Hevea*. HbERF-IXc4 and HbERF-IXc5 are two orthologs of ERF1, which is at the crosstalk of the ethylene and jasmonate signalling pathways^35^. Lastly, ethylene stimulation induced several families of microRNAs^36–38^. This also suggests post-transcriptional regulation by ethylene.

This study also revealed dramatic down-regulation of DEGs related to jasmonic acid (JA) stimulus, carbohydrate and wounding responses. Tapping induces mechanical wounding, which is responsible for jasmonate production. Several upstream regulators down-expressed during TPD occurrence may explain these results. For instance, MYC transcription factors involved in the activation of JA-responsive genes were strongly under-expressed by ethephon^39^. Regulation of *MYC* gene expression by ethephon was confirmed by this study as well as in TPD-affected trees. Several ethylene signalling transcription factors are also known to drive the response to jasmonate. The EIN3 gene, which encodes a primary transcription factor of the ethylene signalling pathway, is induced by both methyl jasmonate and ethylene, and might control JA/ethylene-responsive genes^36,40^. ERFs from groups II and VIII can also more specifically control JA-responsive genes. Contig CL17512Contig1 corresponding to HbERF-IIb2, an ortholog of ORA57 involved in the control of JA biosynthesis^35^, was expressed at a very low level. This confirms previous assumptions that the expression of most genes related to jasmonate synthesis is repressed in TPD-affected trees^18^.

The involvement of oxidative stress in TPD was recently reviewed^2^. This study showed that ethephon stimulation and TPD severity respectively induced the under-expression of 113 and 34 genes, which are related to “oxido/reductase activity” (Suppl. Data 8). Other transcriptomic and proteomic analyses also highlighted that ROS metabolism, the ubiquitin-proteasome pathway and programmed cell death were associated with TPD occurrence^29,41^. The involvement of reactive oxygen species in the latex metabolism and TPD syndrome, and the wounding tolerance of rubber trees, suggest that some HbERFs from group VII, involved in the response to hypoxia, might play a major role in latex production. Several members of three ERF groups (I, VII, VIII) are highly expressed in latex^21^. In TPD-affected trees, the consumption of oxygen by NADH-cytochrome-c-oxidoreductase from lutoids is particularly high^42^. According to these authors, NADH-dependent oxygen consumption is inhibited by superoxide dismutase activity^43^. ERF-VII transcription factors are known to regulate hypoxia-responsive genes. HbERF-VIIa17, an ortholog gene of AtEBP, might play a role in the response to the accumulation of reactive oxygen species occurring during latex regeneration^21^.

To conclude, this study is the first high-throughput RNA sequencing in latex at the onset of ethephon-induced TPD. The overall differentially expressed genes associated with ethephon stimulation and the severity of TPD have been identified and a gene network has been proposed to address this issue. The depression in jasmonate response and ROS-scavenging systems were a common feature observed in latex (this study) and in bark (previous studies). Allelic variation in the sequences of gene expression markers associated with TPD might be exploited on already mapped and phenotyped segregating populations in order to identify the genetic markers associated with phenotypes related to latex production and TPD susceptibility.

## Methods

### Plant material

Latex was collected from 9-year-old trees of clone PB 260 at the Sembawa Research Centre belonging to the Indonesian Rubber Research Institute (Palembang, Sumatra, Indonesia). Trees were tapped for three years using the S/2 d2 harvesting system (semi-spiral and tapping every two days) with or without application of 2.5% ethephon 12 times a year (ET 12/y). The dry cut length (DCL) was monitored after tapping for 3 years. Trees were categorized as slight- (<25% of DCL) or severe-TPD trees (>75%) according to the mean DCL for the last year of tapping. Nine trees were selected for further RNA sequencing: three slight-TPD trees under S/2 d2 (control), three slight-TPD trees under S/2 d2 ET 12/y, and three severe-TPD trees under S/2 d2 ET 12/y.

### Total RNA isolation

For each selected tree, 6 mL of latex was collected in a 15-mL Falcon tube containing 6 ml of 2X alkaline fixing buffer (0.1 M Tris-HCl, 0.3 M LiCl, 10 mM EDTA, 10% SDS pH 9) with constant manual mixing, and immediately deep-frozen in liquid nitrogen. Total RNAs were isolated using the caesium chloride (CsCl) cushion method adapted from Sambrook^44^ by Duan and coll.^36^. Latex samples were thawed in a water bath at 50 °C and transferred to centrifuge tubes. After centrifugation for 30 min at 15,000 *g* at 20 °C, the aqueous fraction was transferred to a 13 mL-tube, then the volume was adjusted to 10 mL with extraction buffer containing 4 M guanidium isothiocyanate, 1% sarcosine, 1% polyvinylpyrrolidone and 1% ß-mercapto-ethanol. After homogenization, the tubes were kept on ice, then centrifuged at 10,000 g at 4 °C for 30 minutes. The supernatant was transferred to a new tube containing 3 mL of 5.7 M CsCl. Ultracentrifugation in a swinging bucket was carried out at 82,705 *g* at 20 °C for 20 hours. The supernatant and caesium cushion were discarded whilst the RNA pellet was washed with 70% ethanol. After 30 minutes of air drying, the pellet was dissolved in 200 µL of sterile water. Although DNA could not cross the caesium cushion for this centrifugation condition, a DNAse treatment was performed using TurboDNAse (Ambion, Life Technologies, Carlsbad, USA). DNA contamination was then checked by PCR amplification using primers of the *Actin* gene including the intron sequence. RNAs were conserved at −80 °C pending further analysis.

#### RNA sequencing and count analysis

The quality and quantity of total RNA samples were checked using the Agilent Bioanalyzer and Quant-iT RiboGreen RNA Assay Kit, respectively. The sequencing technology used was an Illumina Hiseq 2000 (thanks to IG-CNS for allowing us privileged access to carry out sequencing). RNA-seq libraries were produced by the TruSeq_Stranded_mRNA_SamplePrep_Guide_15031047_D protocol (Illumina®, California, U.S.A.) using 2 µg of total RNA. The RNA-seq samples were sequenced in paired-end (PE) mode with a sizing of 260 bp and a read length of 100 bases. Seven samples per Hiseq 2000 lane were analysed using individual bar-coded adapters, generating approximately 60 million PE reads per sample.

Each RNA-seq sample followed the same pipeline of bioinformatics analyses from trimming to the counting of transcript abundance as follows. Read pre-processing criteria included trimming library adapters and performing quality control. The raw data (fastq) were trimmed by the fastx toolkit for Phred Quality Score Qscore >20, read length >30 bases, and the ribosome sequences were removed with the sortMeRNA tool^45^. The mapper Bowtie version 2^46^ was used to align reads against the rubber clone PB 260 reference transcriptome^21^ (with–local option and other default parameters). Reads were counted for the 86,941 contigs. The abundance of each contig was calculated by a local script which parses SAM files and counts only paired-end reads for which both reads map unambiguously one contig, removing multi-hits. According to this procedure, around 95% of PE reads were associated with a contig.

Statistical analyses were carried out with R software (version 3.3.1) and the edgeR package (version1.12.0). Library sizes were normalized using the TMM method. A differential expression analysis was performed using an exact test based on negative binomial distribution using an empirical Bayes estimation of the dispersion, where data were filtered by the default mode proposed by edgeR: genes that did not have at least 1 read after a count per million in at least half of the samples were discarded. To control the FDR, raw p-values were adjusted. We considered as being differentially expressed, those contigs with an adjusted p-value under 0.05, fulfilling criteria for a robust RNA-Seq analysis^47^. All steps of the transcriptomic experiment, from growth conditions to bioinformatic analyses were stored and managed through the CATdb database^48^ (http://tools.ips2.u-psud.fr.fr/CATdb/) Project_ID NGS2014_07_Hevea according to the “minimum information about a high-throughput sequencing experiment” standards.

A principal component analysis (PCA) and heatmap clustering analysis were performed using the R Mixomics and flashClust packages, respectively, after log transformation of the normalized count data. Data scaling and the Euclidean distance were calculated by the Pareto and Ward methods, respectively.

A BINGO network analysis was carried out using Cytoscape 3.4.0 for the DEG groups. DEGs were converted into their respective *Arabidopsis thaliana* orthologs selected according to the best e-values. The At DEGs were implemented to perform gene ontology (GO) enrichment using the BiNGO plugin (Cytoscape© software)^49^ following the procedure by default. Briefly, over-representation of the biological process was assessed when compared to *Arabidopsis thaliana* whole annotation as the reference. A hypergeometric test was used as a statistical test, then multiple testing correction was applied using Benjamini & Hochberg False Discovery Rate (FDR) correction at the significant level of 0.05. The Bingo graph represents the over-represented GO category as nodes. Node size is proportional to the gene number related to the respective category. The colour of the nodes represents the significance of the over-representation, with a colour scale ranging from yellow at a p-value of 0.05 to dark orange at a p-value corresponding to 5 orders of magnitude smaller than the significance level (10^−5^ × 0.05).

### Data deposition

The RNA sequencing data have been submitted to the international repository GEO (Gene Expression Omnibus, Edgard R. *et al*. 2002, http://www.ncbi.nlm.nih.gov/geo) ProjectID GSE101568.

## Electronic supplementary material

Supplementary Data
